# Disordered Eating in a Digital Age: Eating Behaviors, Health, and Quality of Life in Users of Websites With Pro-Eating Disorder Content

**DOI:** 10.2196/jmir.2023

**Published:** 2012-10-25

**Authors:** Rebecka Peebles, Jenny L Wilson, Iris F Litt, Kristina K Hardy, James D Lock, Julia R Mann, Dina LG Borzekowski

**Affiliations:** ^1^The Craig Dalsimer Division of Adolescent MedicineDepartment of PediatricsThe Children's Hospital of Philadelphia, Perelman School of Medicine at The University of PennsylvaniaPhiladelphia, PAUnited States; ^2^Division of Pediatric NeurologyDepartment of PediatricsThe Children's Hospital of Philadelphia, Perelman School of Medicine at The University of PennsylvaniaPhiladelphia, PAUnited States; ^3^Department of PediatricsStanford UniversityStanford, CAUnited States; ^4^Department of NeurologyChildren's National Medical CenterWashington, DCUnited States; ^5^Division of Child and Adolescent PsychiatryDepartment of Psychiatry and Behavioral SciencesStanford University School of MedicinePalo Alto, CAUnited States; ^6^Rice UniversityHouston, TXUnited States; ^7^Department of Health, Behavior and SocietyJohns Hopkins Bloomberg School of Public HealthBaltimore, MDUnited States

**Keywords:** Eating disorder, social network, anorexia nervosa, bulimia nervosa, pro-eating disorder website, pro-anorexia website, pro-bulimia website, pro-ED, pro-ana, pro-mia

## Abstract

**Background:**

Much concern has been raised over pro-eating disorder (pro-ED) website communities, but little quantitative research has been conducted on these websites and their users.

**Objective:**

To examine associations between levels of pro-ED website usage, disordered eating behaviors, and quality of life.

**Methods:**

We conducted a cross-sectional, Internet-based survey of adult pro-ED website users. Main outcomes were Eating Disorder Examination Questionnaire (EDE-Q) and Eating Disorder Quality of Life (EDQOL) scores.

**Results:**

We included responses from 1291 participants; 1254 (97.13%) participants were female. Participants had an average age of 22.0 years and a mean body mass index of 22.1 kg/m^2^; 24.83% (296/1192) were underweight; 20.89% (249/1192) were overweight or obese. Over 70% of participants had purged, binged, or used laxatives to control their weight; only 12.91% (163/1263) were in treatment. Mean EDE-Q scores were above the 90th percentile and mean EDQOL scores were in the severely impaired range. When compared with moderate and light usage, heavy pro-ED website usage was associated with higher EDE-Q global (4.89 vs 4.56 for medium and 4.0 for light usage, *P* < .001) and EDQOL total scores (1.64 vs 1.45 for medium and 1.25 for light usage, *P* < .001), and more extreme weight loss behaviors and harmful post-website usage activities. In a multivariate model, the level of pro-ED website usage remained a significant predictor of EDE-Q scores.

**Conclusions:**

Pro-ED website visitors reported many disordered eating behaviors, although few had been treated. Heavy users reported poorer quality of life and more disordered eating behaviors.

## Introduction

The Internet offers numerous websites that can affect the health of vulnerable users. Of particular concern are pro-eating disorder (pro-ED) website communities (also called pro-anorexia or pro-ana, and pro-bulimia or pro-mia), where individuals may learn about, discuss, and reinforce disordered eating behaviors [1]. Pro-recovery websites promote discussion more related to fighting an eating disorder, although online content can overlap between pro-recovery and pro-ED communities [2].

**Figure 1:**
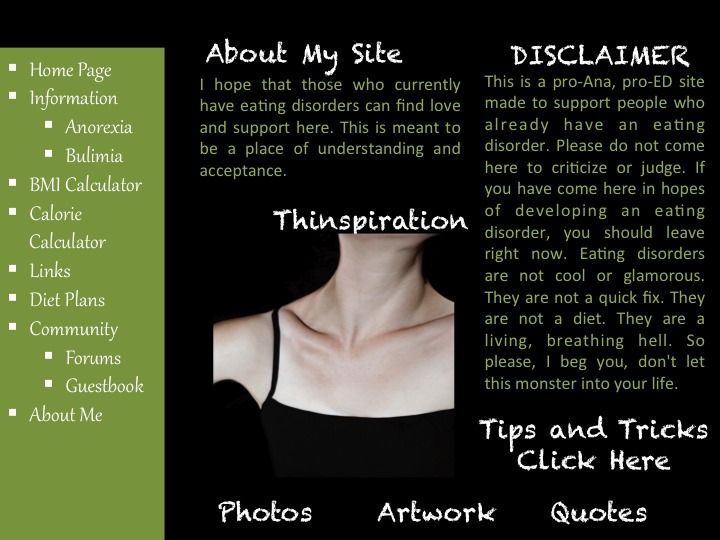
Mock pro-eating disorder website with typical content.

Content on pro-ED websites includes “thinspiration” (images or text for the purpose of inspiring weight loss), techniques to assist in weight loss, and interactive forums [2-6]. Some sites promote eating disorders as a lifestyle choice, offering encouragement for extreme dieting and exercise behaviors and assistance in avoiding detection by family and medical providers. Other websites aim to support visitors at various stages of illness, recognizing the dangers of disordered eating and offering content dedicated to treatment and recovery [4]. See Figure 1 for an example of a mock website.

These websites can have deleterious effects on the user [7-10]. Participants exposed to a pro-ED site for 25 minutes were more likely to show negative affect, perceive themselves as heavier, and exercise or think about weight [11]. Pro-ED visitors have also displayed higher body dissatisfaction, restriction, and bulimic activity than controls [12]. A pilot study of 76 adolescents who had been in treatment for an eating disorder found that over a third had visited pro-ED sites. Practically all (96%) of these pro-ED website users reported learning new weight loss or purging techniques from the sites [1].

Great concern has been raised over pro-ED websites; however, relatively little is known about their users. No study has examined a large group of pro-ED website users from a clinical perspective, nor have associations between disordered eating patterns and escalating levels of site visitation been described. The purpose of this study was to examine the demographics, media use patterns, and eating behaviors of pro-ED website visitors, and the degree to which website usage correlates with disease severity and quality of life.

## Methods

We recruited adult users of websites with pro-ED content via a dedicated link established on identified websites. All protocols were approved by the Stanford University Panel on Medical Research in Human Subjects, Stanford, CA, USA, and were compliant with the Health Insurance Portability and Accountability Act of 1996. Informed consent was obtained from all participants.

### Website Search and Inclusion

We developed a comprehensive new methodology to access users from a broad spectrum of websites with pro-ED content. We searched Google and Yahoo!, entering the keywords “pro-ana” and “pro-mia”. All 700 URL addresses on the first 35 pages of search results for either engine were examined. Websites were included if they displayed any of the following: (1) a declaration that the website was pro-ED, (2) a disclaimer or warning to stay away from the site if the visitor was in recovery or did not have an eating disorder, (3) the term thinspiration, or (4) information on disordered eating behaviors in a framework intended to inform the disorder (tips and techniques). We further searched these websites for links to similar sites. These second-generation websites were also examined and included if they met the above criteria. Additionally, we searched the three most popular open social networking sites as determined by comscore Media Metrix [13] for the keywords pro-ana, ana, pro-mia, and mia. Webrings (collections of related websites) or interest groups found in these searches were included if they met the inclusion criteria and had at least 500 members.

**Figure 2:**
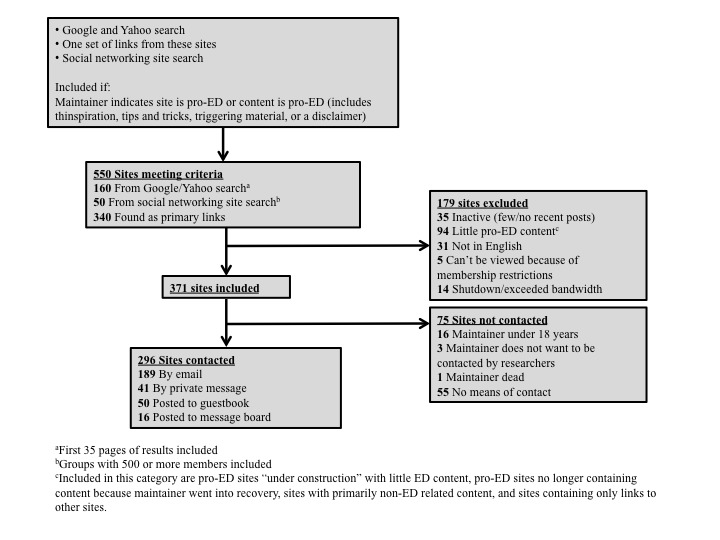
Website search results. ^a^ First 35 pages of results included. ^b^ Groups with 500 or more members included. ^c^ Included in this category are pro-ED (eating disorder) sites under construction with little ED content, pro-ED sites no longer containing content because maintainer went into recovery, sites with primarily non-ED-related content, and sites containing only links to other sites.

Our methods involved no deception. Only English-language sites with active participation or updates within the last 12 months were included. Websites or webrings were excluded if their maintainers indicated that they were less than 18 years of age or did not wish to be involved in research. Figure 2 details the search results.

### Recruitment

We contacted the maintainers of 296 websites, describing the study and its purpose, and asking maintainers to post a link to our study website, which described both the study and the online survey. Participation time was described as 30–40 minutes. On entering the study site, potential participants were asked to confirm that they were over 18 years of age and to give informed consent before being directed to the survey. No incentives were used to encourage participation. No identifiers were collected and no cookies were used. IP addresses were not tracked or limited in their responses. Participants were able to contact the study researchers via email. Survey responses were entered online and automatically captured into a data file.

### Survey

Our online survey contained 193 items, covering demographics, self-reported heights and weights, disordered eating, quality of life, treatment history, overall health, self-harm, and website usage of both pro-ED and pro-recovery websites (see Multimedia Appendix 1). Participants were asked to self-designate their race or ethnicity according to categories defined by the US National Institutes of Health. The Eating Disorder Examination Questionnaire (EDE-Q) assessed the degree of disordered eating and thoughts [14], the core set of the US Centers for Disease Control and Prevention’s (CDC) Health-Related Quality of Life Healthy Days Measure [15] was used as a generic quality-of-life measure, and the Eating Disorder Quality of Life (EDQOL) measure [16] assessed eating disorder-specific health-related quality of life. Finally, we adapted a survey that our team had previously used to evaluate pro-ED and pro-recovery website usage [1]. Participants were also asked if they lied on the survey, and if so, these responses were excluded from analyses. The survey was piloted within all members of the research team to be certain it was functional online prior to fielding the questionnaire.

### Outcome Measures

Main outcome measures were scores on the EDE-Q and EDQOL. Secondary outcomes were the number of unhealthy days on the Healthy Days Measure and common indicators of health in patients with an eating disorder, such as history of admission to hospitalization, osteopenia, or missed menses. The predictor variable for all primary analyses was the level of pro-ED website usage.

### Weight Calculations

We calculated body mass index (BMI) using participants’ self-reported heights and weights, using the equation BMI = weight in kilograms/(height in meters)^2^. Reported BMI values were divided by a median BMI of 21.7 for women and 23.0 for men, based on growth charts developed by the CDC [17], to obtain percentages of median body weight for each participant. Participants were asked for their highest and lowest weights and ages at those times, and definitions of overweight and obesity at those times followed CDC guidelines for children and adults.

### Statistics

We used standard descriptive and frequency testing to describe the data. Associations were assessed using chi-square testing, Student *t* test, Pearson correlations, and analysis of variance, followed by Tukey test for post hoc comparisons. Multivariate regression analyses were used to stratify factors predictive of disordered eating behaviors and impaired quality of life. Responses were not weighted in any manner. Significance level was set at .05. Statistical analysis was conducted using SPSS 19.0 (IBM Corporation, Somers, NY, USA).

## Results

### Response Rate

While nonresponse bias cannot be assessed in anonymous online surveys, we compared the number of visits (hits) to the study website with the number of completed questionnaires to determine a proxy response rate. Over a 10-week period between May and July 2006, there were 3341 hits to our study website and 1456 completed surveys, resulting in a proxy response rate of 43.58%.

Our final analysis included data from 1291 participants, representing 38.64% of initial survey site hits. A total of 227 participants admitted to lying on the survey (15.59% of respondents). Of these, we excluded 165 from analyses because they lied about their age (n = 127) or reported being less than 18 years of age (n = 38). Of those remaining, 70 reported lying about their weight, so we did not analyze their heights and weights. A total of 18 lied about something other than age and weight, with 13 stating that they had done their best to be truthful but had estimated some answers. The remaining 5 lied on very specific portions of the survey, so we excluded answers to those corresponding questions. We discovered no duplicate responses on analyses. One respondent gave nonsensical responses and was excluded.

With regard to the completeness of responses in crucial data on primary predictor and outcome variables, only 1.63% (n = 21) responses were “break-offs” (surveys with less than 50% of questions answered), and another 3.72% (n = 48) were partially complete (50%-80% of questions answered), according to standard definitions of these values in survey reporting as defined by the American Association for Public Opinion Research [18].

### Characteristics of Website Users

Table 1 describes clinical and demographic characteristics reported by participants at the time of the survey, disordered eating behaviors, and views on their own health. Many reported being overweight (282/1188, 23.73%) or obese (406/1188, 34.17%) in the past, with a mean %median BMI at their highest weight of 128.59% (range 70–298, SD 32.71). The mean %median BMI at the lowest reported weight was 88.6% (range 44–233, SD 19.0). While many (800/924, 86.6%) self-diagnosed an eating disorder, 67.62% (873/1291) had never been in treatment, 87.09% (1100/1263) were not currently being treated, and 39.20% (499/1273) had ever had a formal diagnosis of an eating disorder. Underweight participants were more likely to have been treated than normal-weight or overweight respondents (41.6% vs 30.4% or 24.9%, χ^2^_2_ = 18.9, *P* < .001).

**Table 1:** Clinical and demographic characteristics of study participants (N = 1291).

Characteristic		n	%	Mean	SD	Range
Age (years)		1260		22	5.1	18–55
**Gender (n=1291)**						
	Female	1254	97.13			
	Male	37	2.87			
**Ethnicity (n=1291)**						
	White	1100	85.21			
	Hispanic/Latino	63	4.88			
	Asian	35	2.71			
	African American or black	30	2.32			
	American Indian/Alaskan Native	17	1.32			
	Native Hawaiian/Pacific Islander	4	0.31			
	Other	30	2.32			
**Marital status (n=1285)**						
	Married	134	10.38			
	Unmarried	1151	89.16			
**Employment/student status (n=1284)**						
	Student	758	58.71			
	Employed	360	27.89			
	Combined employed/student	37	2.87			
	Unemployed and not a student	129	10.00			
BMI^a^ (kg/m^2^)		1192		22.1	5.7	12.1–59.0
%Median body weight		1192		101.9	26.4	56–272
**Weight class by CDC** ^b^ **criteria (n=1192)**						
	Underweight (BMI <18.5)	296	24.83			
	Normal weight (BMI 18.5–24.9)	647	54.28			
	Overweight (BMI 25–29.9)	148	12.42			
	Obese (BMI ≥30)	101	8.47			
**ED** ^c^ **diagnosis if formally diagnosed (n=498)**						
	Anorexia nervosa	225	45.2			
	Bulimia nervosa	104	20.9			
	EDNOS^d^	151	30.3			
	Binge eating disorder	8	1.6			
	Did not specify	1	0.2			
Age at dieting onset (years)		1236		13	3.7	
Age at ED onset (years)		1031		14.3	3.7	3–40
Disease duration (years)		1006		7.6	5.6	0–43
**Activities in the last month**						
	Counting of calories, fat, or carbohydrates (n = 1265)	1169	92.41			
	Compulsive exercise (n = 1260)	965	76.59			
	Secretive eating (n = 1270)	952	74.96			
**Purging**						
	Ever (n = 1269)	969	76.36			
	Last month (n = 1251)	717	57.31			
	Age at onset	964		15.9	3.8	6-40
**Binge eating**						
	Ever (n = 1269)	1068	83.96			
	Last month (n = 1271)	843	66.33			
	Age at onset	1019		14.5	3.8	3-35
**Laxative use**						
	Ever (n = 1273)	700	54.99			
	Last month (n = 1264)	443	35.05			
	Age at onset	672		17.6	3.9	10-40
**Diet pill use**						
	Ever (n = 1271)	917	72.15			
	Last month (n = 1265)	551	43.66			
	Age at onset	909		17.1	3.5	10-48
	Used >7 times per week	602	65.9			
Age at first ED treatment (years)		402		17.4	3.9	7.0–38.0
**Admission to hospitalization**						
	In past 30 days (n = 1243)	20	1.61			
	Ever (n = 1253)	170	13.57			
**Missed menses in last year (n = 1238)**						
	None	472	38.13			
	<3	248	20.03			
	≥3	358	28.92			
	None, but I am on the pill	148	11.95			
	I have never had a period	12	0.97			
Diagnosis of low bone density (n = 1183)		110	9.30			
**Mental health diagnoses**						
	Depression (n = 1271)	748	58.85			
	Anxiety (n = 1271)	530	41.70			
	ADHD^e^ (n = 1264)	136	10.76			
	Other (n = 1121)	367	32.74			
Ever treated with psychiatric medication (n = 1259)		648	51.47			
**Self-harm**						
	In last 30 days (n = 1270)	454	35.75			
	Ever (n = 1271)	988	77.73			
	Cutting (n = 1291)	841	65.14			
	Burning (n = 1291)	285	22.08			
	Scratching (n = 1291)	558	43.22			
	Other (n = 1291)	351	27.18			
**Views on own health (n = 1257)**						
	Eating disordered by choice	389	30.95			
	Sick	320	25.46			
	Recovering or trying to recover	159	12.65			
	Healthy	140	11.14			
	Other or combination of above	251	19.97			
**Support pro-ED movement (n = 1227)**						
	Completely or very much	539	43.93			
	A little bit or somewhat	487	39.69			
	Not at all	201	16.38			

^a^ Body mass index (kg/m^2^).

^b^ US Centers for Disease Control and Prevention.

^c^ Eating disorder.

^d^ Eating disorder not otherwise specified.

^e^ Attention deficit/hyperactivity disorder

Mean scores on EDE-Q subscale and global scores exceeded the 90th percentile for young adult female norms. Mean scores on subscales were 4.56 (SD 1.25, 95–99 percentile) for Restraint, 3.83 (SD 1.29, 95–99 percentile) for Eating Concern, 5.18 (SD 0.94, 90–95 percentile) for Shape Concern, and 4.86 (SD 1.07, 95–99 percentile) for Weight Concern. The EDE-Q global score showed significant pathology with a mean of 4.61 (SD 0.96, 95–99 percentile).

On the CDC Healthy Days Measure, 5.23% (67/1280) indicated their health was poor, 24.14% (309/1280) fair, 39.61% (507/1280) good, 22.42% (287/1280) very good, and 8.59% (110/1280) excellent. Despite these encouraging answers, participants reported a mean of 6.5 (SD 7.8) physically unhealthy days in the past month, 18.4 mentally unhealthy days (SD 9.9), and 21.1 (SD 9.9) unhealthy days overall. On average, participants reported 7.5 (SD 8.3) days in which their activity was limited in the last month. Moreover, 24.00% (248/1033) reported they had to stop school, and 17.49% (192/1098) needed to stop working in the past because of their eating disorder. As well, 38.06% (427/1122) spent less time on their school or work, 36.10% (422/1169) spent less time in recreational activities, and 58.70% (725/1235) spent less time with friends over the past month, owing to their eating disorder.

EDQOL subscales were all in the severely impaired range, with participants demonstrating a mean psychological score of 2.78 (SD 0.74), physical/cognitive score of 1.97 (SD 0.87), financial score of 0.55 (SD 0.82), and work/school score of 0.63 (SD 0.81). Overall, the global quality-of-life score on this measure was 1.49 (SD 0.60), also in the severely impaired range.

**Figure 3:**
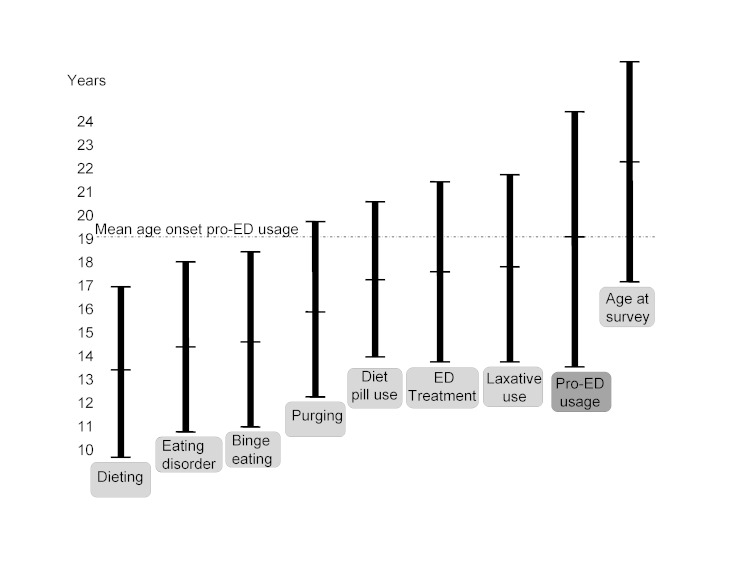
Self-reported age of onset of dieting behaviors and pro-eating disorder (ED) website usage. Mean age of onset of ED-related activities indicated by center horizontal bar, with vertical standard deviation error bars. Horizontal dashed line represents mean onset of pro-ED usage.

The reported average age at onset of visiting pro-ED websites was higher than the reported age at onset of other disordered eating behaviors and years after participants felt their eating disorder had begun (Figure 3).

### Website Usage

When asked from which sources they obtained the most eating disorder information in the last 30 days, 86.65% (1077/1243) of participants reported a website, either pro-ana (611/1243, 49.16%), pro-mia (4.42%, 55/1243), pro-recovery (44/1243, 3.54%), or a general health website (27/1243, 2.17%), and the Internet in general (340/1243, 27.35%). Only a few (39/1243, 3.14%) reported consulting books, and only 1.21% (15/1243) reported consulting physicians. The dominance of the Internet for this population was not surprising, given that 35.73% (453/1268) spent more than 4 hours on the Internet daily, 34.94% (443/1268) spent 2–4 hours each day, and 23.11% (293/1268) spent 1–2 hours each day. Only 6.23% (79/1268) spent less than 1 hour on the Internet daily.

Most participants learned about pro-ED sites by chance (403/1228, 32.82%) or by reading about them (308/1228, 25.08%). A third (466/1269, 36.72%) indicated that they had visited a pro-recovery site in the past 30 days, and 62.20% (790/1270) had ever visited such a site. Table 2 presents additional details regarding the online activities and feelings of this population about pro-ED and pro-recovery websites.

**Table 2:** Online activities and feelings of the study overall population on pro-eating disorder (pro-ED) websites and of the subgroup using pro-recovery websites.

			Pro-ED websites			Pro-recovery websites		
			n	%	Mean (SD)	n	%	Mean (SD)
**Reported activities or feelings**								
	Age first visited (years)		1218		18.9 (5.5)	735		19.3 (4.8)
	Hours/week in last 30 days		1147		10.5 (18.2)	681		2.3 (6.8)
	Have their own site		212	17.24		42	5.7	
	**Felt supported by website in last 30 days**		n = 1210			n = 692		
		Extremely	351	29.01		46	6.6	
		Very	385	31.82		66	9.5	
		Somewhat	254	20.99		151	21.8	
		A little	115	9.50		183	26.4	
		Not at all	105	8.68		246	35.5	
	**Found a community like self on website**		n = 1220			n = 705		
		Completely	412	33.77		59	8.4	
		Very much	335	27.46		78	11.1	
		Somewhat	201	16.48		116	16.5	
		A little	152	12.46		203	28.8	
		Not at all	120	9.84		249	35.3	
	**Most important reason for accessing site in last 30 days**		n = 1211			n = 658		
		Motivation for weight loss	447	36.91		89	13.5	
		Weight loss tips	192	15.85		54	8.2	
		Meeting people	63	5.20		13	2.0	
		Tips on hiding eating disorder	48	3.96		32	4.9	
		Curiosity	95	7.84		220	33.4	
		Support	318	26.26		190	28.9	
		Help with recovery	2	0.17		22	3.3	
	**Activity at sites**		n = 1289			n = 790		
		Read posts	1120	86.89		534	67.6	
		Visited chat room	307	23.82		89	11.3	
		Posted messages	696	54.00		167	21.1	
		Read diaries or blogs	913	70.83		358	45.3	
		Created my own diary or blog	433	33.59		86	10.9	
		Learned about weight loss methods	789	61.21		218	27.6	
		Learned about diet pills, laxatives, or weight loss supplements	621	48.18		197	24.9	
		Learned about places to purchase new diet pills, laxatives, or weight loss supplements	294	22.81		80	10.1	
	**Post-website use activity**					n = 790		
		Changed eating habits	671	54.55		137	18.6	
		Used new diet pills, laxatives, or weight loss supplements in past 30 days	210	17.21		37	5.2	
		Used new diet pills, laxatives, or weight loss supplements ever	454	37.09		128	17.9	
		New weight loss or purging methods in past 30 days	391	32.26		99	13.6	
		New weight loss or purging methods ever	748	60.86		224	30.7	
		Self-harm in past 30 days	68	5.60		40	5.6	
		Self-harm ever	180	14.65		95	12.9	
**Site components that were motivational for continuing eating disorder or recovery**								
	Photographs and artwork		1097	89.70		426	58.8	
	Forum postings and chat rooms		1040	85.60		552	77.0	
	Diet and exercise information		1086	89.16		499	69.3	
	Diaries, journal entries, and blogs		1017	83.50		551	76.3	

### Website Usage Level and Outcomes

We grouped participants based on the frequency and duration of pro-ED website usage. Light users (n = 199, 16.1%) were those who reported less than 1 month of website usage regardless of frequency, or 1–12 months of usage with a frequency of less than once a month. Heavy users (n = 513, 41.5%) were those who used the websites at least daily and had visited them for 12 or more months. All other participants were considered medium users (n = 525, 42.4%). Mean hours/week of website usage were 3.02 (SD 4.8), 7.8 (SD 12.8), and 16.1 (SD 23.7, *P* < .001) for light, medium, and heavy users, respectively. Differences between groups in outcomes are presented in Table 3.

**Table 3:** Pro-eating disorder (pro-ED) website usage and associated eating disorder activities and outcomes.

Characteristic		Total	Light (n = 199)	Medium (n = 525)	Heavy (n = 513)	χ^2^/*F*	*df*	*P* value
Age at survey (years), mean		22.0	23.1	22	21.6	6.1^a^	2	.008
Current %median body weight, mean		101.9	106.3	101.4	100.5	3.5^b^	2	.006
**Eating Disorder Examination Questionnaire score, mean**								
	Restraint	4.56	3.86	4.55	4.86	50.4^c^	2	≤.001
	Eating concerns	3.83	3.24	3.71	4.17	43.8^c^	2	≤.001
	Shape concerns	5.18	4.65	5.15	5.4	49.1^c^	2	≤.001
	Weight concerns	4.86	4.27	5.82	5.13	51.6^c^	2	≤.001
	Global	4.61	4	4.56	4.89	69.2^c^	2	≤.001
**Healthy days (in last 30 days)**								
	Overall health poor (n = 1280)	67 (5.23%)	4.5	3.8	7.2	29.2^c^	8	≤.001
	Overall health excellent (n = 1280)	110 (8.59%)	14.1	9	6.4	29.2^c^	8	≤.001
	Unhealthy physical days, mean	6.5	5.3	5.8	7.7	11.2^c^	2	≤.001
	Unhealthy mental days, mean	18.4	16.6	17.6	20	11.2^c^	2	≤.001
	Sum unhealthy days, mean	21.1	19.1	20.4	22.6	11.2^c^	2	≤.001
	Days of limited activities, mean	7.5	7	7.1	8.3	3.0^d^	2	.05
**Eating Disorder Quality of Life score, mean**								
	Psychological	2.78	2.43	2.74	2.95	39.0^c^	2	≤.001
	Physical	1.97	1.60	1.92	2.17	33.7^c^	2	≤.001
	Financial	0.55	0.47	0.49	0.64	5.3^a^	2	.005
	Work/school	0.63	0.43	0.59	0.76	13.1^c^	2	≤.001
	Total	1.50	1.25	1.45	1.64	34.7^c^	2	≤.001
Amenorrhea (n = 1082)		367 (33.92%)	25.1	28.7	42.4	29.2^c^	4	≤.001
Low bone mineral density (n = 1183)		110 (9.30%)	8.9	8.7	10.1	.574	2	.750
Age started dieting (years), mean		13.3	14.3	13.5	12.6	16.7^c^	2	≤.001
Age eating disorder began (years), mean		14.3	14.7	14.6	13.9	4.2^b^	2	.015
Disease duration (years), mean		7.6	8.1	7.5	7.6	.564	2	.569
History of eating disorder treatment (n = 1291)		419 (32.46%)	23.6	28.0	39.2	22.5^c^	2	≤.001
History of hospitalization (n = 1253)		170 (13.57%)	6.6	12.3	17.3	15.1^c^	2	.001
Age first binged (years), mean		14.5	14.7	14.5	14.5	.238	2	.788
**Binge eating**								
	Last 30 days (n = 1271)	843 (66.33%)	66	66.5	65.8	.051	2	.975
	Ever (n = 1272)	1068 (83.96%)	80.2	81.1	88.3	12.4^a^	2	.002
Age first purged (years), mean		15.9	16.4	16.2	15.6	4.5^b^	2	.012
**Purging**								
	Last 30 days (n = 1251)	717 (57.31%)	48.5	53.2	65.1	22.3^c^	2	≤.001
	Ever (n = 1269)	969 (76.36%)	66.3	72.8	84.4	32.9^c^	2	≤.001
Age first used laxatives (years), mean		17.6	18.4	17.9	17.2	4.3^b^	2	.014
**Laxative use**								
	Last 30 days (n = 1264)	443 (35.05%)	28.6	32.8	39.8	9.8^a^	2	.007
	Ever (n = 1273)	700 (54.99%)	45.5	51.5	62.8	22.3^c^	2	≤.001
Age first used diet pills (years), mean		17.1	18.2	17.3	16.7	8.4^c^	2	≤.001
**Diet pill use**								
	Last 30 days (n = 1270)	551 (43.66%)	22.7	42.1	53.6	55.8^c^	2	≤.001
	Ever (n = 1271)	917 (72.15%)	53.5	72.0	79.7	48.9^c^	2	≤.001
Excessive exercise last 30 days (n = 1260)		965 (76.59%)	68.4	74.5	82.3	18.0^c^	2	≤.001
**Self-injury**								
	Last 30 days (n = 1270)	454 (35.75%)	26.3	34.0	41.9	16.9^c^	2	≤.001
	Ever (n = 1271)	988 (77.73%)	66.2	76.7	83.0	23.8^c^	2	≤.001
Age first visited a pro-ED site (years), mean		18.9	21.3	19.5	17.5	40.5^c^	2	≤.001
**Eating-disordered activity at sites**								
	Learned about weight loss methods (n = 1289)	789 (61.21%)	61.3	66.5	61.6	3.2	2	.200
	Learned about diet pills or laxatives (n = 1289)	621 (48.18%)	35.7	52	53.6	19.8^c^	2	≤.001
	Learned about places to purchase new diet pills, laxatives, or weight loss supplements (n = 1289)	294 (22.81%)	15.1	22.5	28.1	14.1^c^	2	.001
**Post-website use activity resulting from visiting pro-ED websites**								
	Eating habits have changed (n = 1230)	671 (54.55%)	40.3	58.2	56.1	19.3^c^	2	≤.001
	Used new diet pills, laxatives, or weight loss supplements in past 30 days (n = 1220)	210 (17.21%)	6.2	17.5	20.9	21.8^c^	2	≤.001
	Used new diet pills, laxatives, or weight loss supplements ever (n = 1224)	454 (37.09%)	17.9	34.3	46.9	54.0^c^	2	≤.001
	Used new weight loss or purging methods in past 30 days (n = 1212)	391 (32.26%)	28.4	34.6	31.3	2.7	2	.253
	Used new weight loss or purging methods ever (n = 1229)	748 (60.86%)	45.1	64	63.4	23.6^c^	2	≤.001
	Self-harm in past 30 days (n = 1214)	68 (5.60%)	1.6	5.4	7.1	8.1^b^	2	.017
	Self-harm ever (n = 1229)	180 (14.65%)	6.6	13	19.1	19.6^c^	2	≤.001
Host own pro-ED website (n = 1230)		212 (17.24%)	3.6	11.9	27.8	76.1^c^	2	≤.001
Completely support pro-ED (n = 1227)		329 (26.81%)	7.3	25	36.1	135.8^c^	8	≤.001

^a^ *P* < .01; in analysis of variance (ANOVA) testing: post hoc differences between light and medium and light and heavy.

^b^ *P* < .05; in ANOVA testing: post hoc differences between light and heavy for %median body weight and age when first used laxatives to control weight, between medium and heavy for age when eating disorder began; between light and heavy and medium and heavy for age at first purge.

^c^ P ≤ .001; in ANOVA testing: post hoc differences between all groups for all Eating Disorder Examination Questionnaire scores, Eating Disorder Quality of Life (EDQOL) psychological, physical, and total scores, age started dieting, and age first used pro-ED sites; between light and medium and light and heavy for age first used diet pills; between heavy and light and heavy and medium for EDQOL financial and work/school subscores, and unhealthy physical, mental, and sum days.

### Predictors of Disordered Eating and Quality of Life Impairment

To estimate models predicting EDE-Q and EDQOL global scores, we first examined colinearity among the variables of interest. Age and duration of disease were strongly and significantly correlated (*r* = .76), as were %median body weight and %median BMI at highest (*r* = .74) and lowest (*r* = .77) weights. While other variables were correlated, no others were at the point of colinearity. Therefore, we entered age, %median body weight, level of pro-ED website usage, and EDE-Q and EDQOL global scores into our multivariate models.

Significant predictors of EDE-Q scores were EDQOL global score (beta = .56, *P* < .001) and higher pro-ED usage level (beta = .19, *P* < .001), predicting 40% of the variance (*F*_4,1116_= 185.2, *P*< .001). Significant predictors of EDQOL scores were EDE-Q scores (beta = .59, *P* < .001) and age (beta = –.07, *P* < .005), while pro-ED usage level was not significant (beta = .04, *P* = .13). This variable set explained 38% of the variance in EDQOL scores (*F*_4,1116_ = 167.4, *P* < .001).

## Discussion

This was the largest and most comprehensive study of adult pro-ED website visitors to date. Most are normal-weight young women, who report multiple extreme weight control behaviors, yet have never been in formal eating disorder treatment. This study challenges presumptions about both pro-ED website users and eating disordered individuals in general.

Our results show a clear association between the level of pro-ED website usage and both disordered eating and quality of life. Pro-ED website usage remains an important predictor of EDE-Q scores even when other commonly reported predictors are considered. Heavy users of pro-ED websites differ significantly from light users; of particular concern are those who spend, on average, around 16 hours per week on these websites. More website usage was strongly associated with higher levels of disordered eating on the EDE-Q and more severe impairment on the EDQOL. Usage level was also incrementally associated with younger age at dieting onset, various disordered eating behaviors, and most harmful post-website use activities, such as diet pill use, weight loss techniques, or self-injury. Nearly a third of heavy website users hosted their own pro-ED website and supported pro-ED as a movement. Website usage was also strongly associated with treatment and hospitalization rates. This was a cross-sectional study, so causality cannot be inferred and may simply reflect the degree of illness in heavy website users. However, the reported average age at onset of visiting pro-ED websites was higher than the reported age at onset of other disordered eating behaviors, and years after participants felt their eating disorder had begun. This suggests a disease progression in which website visitation is a later consequence, and not an earlier cause.

These participants differ from the media’s typical image of underweight teenaged pro-ED users, as over half of participants had been overweight or obese, and a fifth were overweight or obese at the time of the survey. Participants displayed high levels of eating disorder pathology and impaired quality of life on validated measures, consistent with those previously reported for homogeneous populations of more strictly defined anorexia nervosa patients [14,16,18]. Pro-ED website users self-reported dangerous behaviors including purging, laxative use, compulsive exercise, and diet pill use. They also reported high levels of eating disorder sequelae and comorbidities, with nearly 50% reporting menstrual irregularities in the last year. Psychiatric diagnoses were prevalent, with 59% reporting depression, 42% anxiety, 52% a history of treatment with psychiatric medication, and over 75% with a history of self-injury.

Despite the high level of pathology and the majority self-diagnosing an eating disorder, only one-third of participants had ever received formal care for their disordered eating. These findings suggest inadequate screening and diagnosis of eating disorders, and that pro-ED website users are seeking support online instead of with a traditional health model. This is a complex phenomenon, and it has been suggested that pro-ED websites may provide a safe, nonjudgmental, and possibly therapeutic interactive environment [19]. However, they have also been noted to offer advice on managing eating disorders, and may subvert mainstream medical care systems by failing to portray eating disorders as negative conditions requiring professional treatment [20]. Our finding that treatment was much less common in normal-weight, overweight, or obese participants raises the question of whether disordered eating may be more likely to be missed in normal and overweight populations, and that these individuals in particular may seek online support.

Respondents reported an astonishingly high 21.1 unhealthy days in the past month (a normal value is 6 unhealthy days in the past 30 days, with diabetic people averaging 11, and breast cancer patients averaging 12) [15]. Participants also had severe levels of impairment on a disease-specific measure, the EDQOL. These results also indicate the need for further prospective study on quality of life and disordered eating, as they appear to be strongly associated.

Online survey methods are new and developing. This comprehensive study design successfully obtained representation from a wide spectrum of websites meeting specific inclusion criteria based on pro-ED content. Engaging website designers and maintainers to incorporate dedicated links to our study allowed us to include over 1200 participants over a relatively short study period. We also assessed participant truthfulness, which allowed us to further improve the quality of data analyzed. Finally, studying participants via the Internet not only offers a population more diverse than many previously reported clinical samples, but also circumvents the ethical problem of inadvertently introducing potentially harmful website content to a population previously naïve to pro-ED sites.

This study has some important limitations. First, respondents were queried about activities that began many years prior to the study, rendering the possibility of recall bias. Second, this was a convenience sample of users who chose to participate, potentially resulting in a selection bias. Third, all online surveys are limited by an inability to meet with participants to verify responses, although prior studies have noted that truthfulness is actually increased using online survey tools when discussing sensitive topics [21]. Fourth, our survey design did not involve a method of access control and thus could have had duplicate responses from the same IP address. Finally, because so few respondents had received treatment, it is possible that the participants underreported or had not yet identified some medical outcomes.

These findings highlight the need to consider the Internet more often as a vehicle for intervention and study, as it offers easy accessibility to users. If appropriate and helpful interventions are developed, they have the potential to reach many people beyond traditional treatment center walls. Users of pro-ED content websites visit them not just for motivation for weight loss or specific dieting tips, but also for emotional support. Pro-recovery websites do not resonate as well with these users, indicating that the medical community needs to listen to online health seekers to determine whether there are self-help sites or educational modules that this population would find meaningful.

Websites with pro-ED content may play both supportive and harmful roles for those struggling with disordered eating. While the content of these sites may affect the eating behaviors of website visitors, the extent of website usage appears to have a more central role in eating behaviors, weight concerns, and quality of life. Moreover, these findings confirm that many with significantly disordered eating, medical complications, psychiatric comorbidity, and severely impaired quality of life are not accessing traditional care and do not fit conventional eating disorder models. They seek support from a Web-based peer group, which poses both potential harms and opportunities for interventions within these online communities. It is critical that future studies comprehensively address possibilities for intervention and improved relationships with these forums, in order to advance our treatment and screening procedures into an online age.

## Multimedia Appendix 1

Online survey. Note: The survey was one continuous online page.

## Abbreviations

BMI: body mass index
CDC: Centers for Disease Control and Prevention
EDE-Q: Eating Disorder Examination Questionnaire
EDQOL: Eating Disorder Quality of Life
pro-ED: pro-eating disorder
