# Supplementary material for: Disordered Eating in a Digital Age: Eating Behaviors, Health, and Quality of Life in Users of Websites With Pro-Eating Disorder Content
Source: J Med Internet Res. 2012 Oct 25;14(5):e148. doi: 10.2196/jmir.2023 (PMC3510745; doi:10.2196/jmir.2023)
Supplement: Supplementary file 1 [file jmir_v14i5e148_app1.pdf]

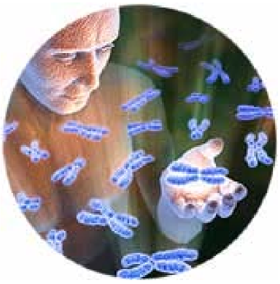

Pro-eating disorder website visitors survey

* = Required

Note: This is an ANONYMOUS questionnaire. There will be no way to link this questionnaire with you as an individual.

1. Have you read the consent form, do you understand the study, and are you willing to participate by answering this questionnaire?*

   ◉ Yes ○ No

2. From which website were you directed to complete this survey?*

3. Today's date (mm/dd/yy):*

Tell us about yourself

4. Date of birth (month and year)*

5. Gender:*

   ○ Male ○ Female

6. Race/Ethnicity
   - ○ White
   - ○ Hispanic/Latino
   - ○ Asian
   - ○ American Indian/Alaskan native
   - ○ Black or African American
   - ○ Native Hawaiian or other Pacific Islander
   - ○ Other/more than one (Please Specify)

7. Marital Status
   - ○ Never married
   - ○ Married (first marriage)
   - ○ Divorced or widowed and presently remarried
   - ○ monogamous relationship, living with partner (but not married)
   - ○ Monogamous relationship, not living with partner
   - ○ Divorced and not presently married
   - ○ Widowed and not presently married
   - ○ Other (Please Specify)

8. What is your primary role?
   - ○ Wage earner, full-time
   - ○ Wage earner, part-time
   - ○ Student, full-time
   - ○ Student, part-time
   - ○ Homemaker
   - ○ Unemployed

Other (Please Specify)

Your stats

9. Current height (in feet and inches)

10. Current weight (in pounds):

11. Your highest weight (in pounds):

12. How tall were you at your highest weight (in feet and inches)?

13. How old were you at your highest weight (in years)?

14. Your lowest weight at your current height (in pounds):

15. How old were you then (in years)?

How is your health?

16. Would you say that in general your health is
Excellent Very good Good Fair Poor

17. Now thinking about your physical health, which includes physical illness and injury, for how many days during the past 30 days was your physical health not good?

18. Now thinking about your mental health, which includes stress, depression, and problems with emotions, for how many days during the past 30 days was your mental health not good?

19. During the past 30 days, for about how many days did poor physical or mental health keep you from doing your usual activities, such as self-care, work, or recreation?

Eating Disorder Quality of Life (EDQOL) Scale

Copyright (c) 2005. Neuropsychiatric Research Institute and Scott Engel.

INSTRUCTIONS: Please answer the following statements according to how well they describe you in the last 30 days. Please be as open as possible. There are no right or wrong answers. Please mark the appropriate response. For those items that do not apply to you, please leave them blank.

Psychological

20. In the past 30 days how often has your eating/weight resulted in you feeling embarrassed or "different"?
Never Rarely Sometimes Often Always

21. In the past 30 days how often has your eating/weight made you feel worse about yourself?
Never Rarely Sometimes Often Always

22. In the past 30 days how often has your eating/weight made you not want to be with people?
Never Rarely Sometimes Often Always

23. In the past 30 days how often has your eating/weight resulted in you believing that you will never get better?
○ Never ○ Rarely ○ Sometimes ○ Often ○ Always

24. In the past 30 days how often has your eating/weight made you feel lonely?
○ Never ○ Rarely ○ Sometimes ○ Often ○ Always

25. In the past 30 days how often has your eating/weight resulted in less interest or pleasure in activities?
○ Never ○ Rarely ○ Sometimes ○ Often ○ Always

26. In the past 30 days how often has your eating/weight led you to not care about yourself?
○ Never ○ Rarely ○ Sometimes ○ Often ○ Always

27. In the past 30 days how often has your eating/weight made you feel odd, weird, or unusual?
○ Never ○ Rarely ○ Sometimes ○ Often ○ Always

28. In the past 30 days how often has your eating/weight resulted in avoiding eating in front of others?
○ Never ○ Rarely ○ Sometimes ○ Often ○ Always

Physical / Cognitive

29. In the past 30 days how often has your eating/weight caused cold hands or feet?
○ Never ○ Rarely ○ Sometimes ○ Often ○ Always

30. In the past 30 days how often has your eating/weight caused frequent headaches?
○ Never ○ Rarely ○ Sometimes ○ Often ○ Always

31. In the past 30 days how often has your eating/weight caused weakness?
○ Never ○ Rarely ○ Sometimes ○ Often ○ Always

32. In the past 30 days how often has your eating/weight affected your ability to pay attention when you wanted to?
○ Never ○ Rarely ○ Sometimes ○ Often ○ Always

33. In the past 30 days how often has your eating/weight affected your ability to comprehend some verbal and written information?
○ Never ○ Rarely ○ Sometimes ○ Often ○ Always

34. In the past 30 days how often has your eating/weight reduced your ability to concentrate?
○ Never ○ Rarely ○ Sometimes ○ Often ○ Always

Financial

35. In the past 30 days how often has your eating/weight led to problems with treatment provider(s) regarding cost of treatment?
○ Never ○ Rarely ○ Sometimes ○ Often ○ Always

36. In the past 30 days how often has your eating/weight led to you having difficulty paying monthly bills?
○ Never ○ Rarely ○ Sometimes ○ Often ○ Always

37. In the past 30 days how often has your eating/weight resulted in significant financial debt?
○ Never ○ Rarely ○ Sometimes ○ Often ○ Always

38. In the past 30 days how often has your eating/weight led to the need to spend money from savings or use your credit card frequently?
○ Never ○ Rarely ○ Sometimes ○ Often ○ Always

39. In the past 30 days how often has your eating/weight resulted in the need to borrow money?
○ Never ○ Rarely ○ Sometimes ○ Often ○ Always

Work / School

40. In the past 30 days how often has your eating/weight led to a leave of absence from work?
○ Never ○ Rarely ○ Sometimes ○ Often ○ Always

41. In the past 30 days how often has your eating/weight led to low grades?
○ Never ○ Rarely ○ Sometimes ○ Often ○ Always

42. In the past 30 days how often has your eating/weight resulted in reduced hours at work?
○ Never ○ Rarely ○ Sometimes ○ Often ○ Always

43. In the past 30 days how often has your eating/weight resulted in you losing a job or dropping out of school?
○ Never ○ Rarely ○ Sometimes ○ Often ○ Always

44. In the past 30 days how often has your eating/weight led to failure in a class or classes?
○ Never ○ Rarely ○ Sometimes ○ Often ○ Always

Eating Disorder Examination-Questionnaire Version (EDE-Q)

Fairburn and Beglin, 1994

Instructions: the following questions are concerned with the past four weeks (28 days) only. Please read each question carefully. Please answer all the questions.

Questions 47-59: Please mark the appropriate response. Remember that the questions only refer to the past four weeks (28 days).

45. On how many of the past 28 days have you been deliberately TRYING to limit the amount of food you eat to influence your shape or weight (whether or not you have succeeded)?

- ○ No days
- ○ 1-5 days
- ○ 6-12 days
- ○ 13-15 days
- ○ 16-22 days
- ○ 23-27 days
- ○ Every day

46. On how many of the past 28 days have you gone for long periods of time (8 waking hours or more) without eating anything at all in order to influence your shape or weight?

- ○ No days
- ○ 1-5 days
- ○ 6-12 days
- ○ 13-15 days
- ○ 16-22 days
- ○ 23-27 days
- ○ Every day

47. On how many of the past 28 days have you TRIED to exclude from your diet any foods that you like in order to influence your shape or weight (whether or not you have succeeded)?

- ○ No days
- ○ 1-5 days
- ○ 6-12 days
- ○ 13-15 days
- ○ 16-22 days
- ○ 23-27 days
- ○ Every day

48. On how many of the past 28 days have you TRIED to follow definite rules regarding your eating (for example, a calorie limit) in order to influence your shape or weight (whether or not you have succeeded)?

- ○ No days
- ○ 1-5 days
- ○ 6-12 days
- ○ 13-15 days
- ○ 16-22 days
- ○ 23-27 days
- ○ Every day

49. On how many of the past 28 days have you had a definite desire to have an EMPTY stomach with the aim of influencing your shape or weight?

- ◯ No days
- ◯ 1-5 days
- ◯ 6-12 days
- ◯ 13-15 days
- ◯ 16-22 days
- ◯ 23-27 days
- ◯ Every day

50. On how many of the past 28 days have you had a definite desire to have a TOTALLY FLAT stomach?

- ◯ No days
- ◯ 1-5 days
- ◯ 6-12 days
- ◯ 13-15 days
- ◯ 16-22 days
- ◯ 23-27 days
- ◯ Every day

51. On how many of the past 28 days has thinking about FOOD, EATING OR CALORIES made it difficult to concentrate on things you are interested in (for example, working, following a conversation, or reading)?

- ◯ No days
- ◯ 1-5 days
- ◯ 6-12 days
- ◯ 13-15 days
- ◯ 16-22 days
- ◯ 23-27 days
- ◯ Every day

52. On how many of the past 28 days has thinking about SHAPE OR WEIGHT made it very difficult to concentrate on things you are interested in (for example, working, following a conversation, or reading)?

- ◯ No days
- ◯ 1-5 days
- ◯ 6-12 days
- ◯ 13-15 days
- ◯ 16-22 days
- ◯ 23-27 days
- ◯ Every day

53. On how many of the past 28 days have you had a definite fear of losing control over eating?

- ◯ No days
- ◯ 1-5 days
- ◯ 6-12 days
- ◯ 13-15 days
- ◯ 16-22 days
- ◯ 23-27 days
- ◯ Every day

54. On how many of the past 28 days have you had a definite fear that you might gain weight?

- ◯ No days
- ◯ 1-5 days
- ◯ 6-12 days
- ◯ 13-15 days
- ◯ 16-22 days
- ◯ 23-27 days
- ◯ Every day

55. On how many of the past 28 days have you felt fat?

- ◯ No days
- ◯ 1-5 days
- ◯ 6-12 days

- 13-15 days
- 16-22 days
- 23-27 days
- Every day

56. On how many of the past 28 days have you had a strong desire to lose weight?

- No days
- 1-5 days
- 6-12 days
- 13-15 days
- 16-22 days
- 23-27 days
- Every day

Questions 60-65: Please mark the appropriate response. Remember that the questions only refer to the past four weeks (28 days).

57. Over the past 28 days, how many TIMES have you eaten what other people would regard as an UNUSUALLY LARGE AMOUNT OF FOOD (given the circumstances)?

58. ....On how many of these times did you have a sense of having lost control over your eating (at the time you were eating)?

59. Over the past 28 days, on how many DAYS have such episodes of overeating occurred (i.e., you have eaten an unusually large amount of food AND have had a sense of loss of control at the time)?

60. Over the past 28 days, how many TIMES have you made yourself sick (vomit) as a means of controlling your shape or weight?

61. Over the past 28 days, how many TIMES have you taken laxatives as a means of controlling your shape or weight?

62. Over the past 28 days, how many TIMES have you exercised in a "driven" or "compulsive" way as a means of controlling your weight, shape or amount of fat, or to burn off calories?

Questions 66-68: Please mark the appropriate response. PLEASE NOTE THAT FOR THESE QUESTIONS THE TERM "BINGE EATING" MEANS eating what others would regard as an unusually large amount of food for the circumstances, accompanied by a sense of having lost control over eating.

63. Over the past 28 days, on how many days have you eaten in secret (ie, furtively)? ...Do not count episodes of binge eating.

- No days
- 1-5 days
- 6-12 days
- 13-15 days
- 16-22 days
- 23-27 days
- Every day

64. On what proportion of the times that you have eaten have you felt guilty (felt that you've done wrong) because of its effects on your shape or weight?...Do not count episodes of binge eating.

- None of the times
- A few of the times
- Less than half
- Half of the times

- More than half
- Most of the time
- Every time

65. Over the past 28 days, how concerned have you been about other people seeing you eat? ...Do not count episodes of binge eating

- 0- Not at all
- 1
- 2- Slightly
- 3
- 4- Moderately
- 5
- 6- Markedly

Questions 69-75: Please mark the appropriate response. Remember that the questions only refer to the past four weeks (28 days).

66. Over the past 28 days has your WEIGHT influenced how you think about (judge) yourself as a person?

- 0- Not at all
- 1
- 2- Slightly
- 3
- 4- Moderately
- 5
- 6- Markedly

67. Over the past 28 days has your SHAPE influenced how you think about (judge) yourself as a person?

- 0- Not at all
- 1
- 2- Slightly
- 3
- 4- Moderately
- 5
- 6- Markedly

68. Over the past 28 days how much would it have upset you if you had been asked to weigh yourself once a week (no more, or less, often) for the next four weeks?

- 0- Not at all
- 1
- 2- Slightly
- 3
- 4- Moderately
- 5
- 6- Markedly

69. Over the past 28 days how dissatisfied have you been with your WEIGHT?

- 0- Not at all
- 1
- 2- Slightly
- 3
- 4- Moderately
- 5
- 6- Markedly

70. Over the past 28 days how dissatisfied have you been with your SHAPE?

- 0- Not at all
- 1
- 2- Slightly
- 3
- 4- Moderately
- 5

6- Markedly

71. Over the past 28 days how uncomfortable have you felt seeing your body (for example, seeing your shape in the mirror, in a shop window reflection, while undressing or taking a bath or shower)?

- 0- Not at all
- 1
- 2- Slightly
- 3
- 4- Moderately
- 5
- 6- Markedly

72. Over the past 28 days how uncomfortable have you felt about OTHERS seeing your shape or figure (for example, in communal changing rooms, when swimming, or wearing tight clothes)?

- 0- Not at all
- 1
- 2- Slightly
- 3
- 4- Moderately
- 5
- 6- Markedly

Medical

73. Have you been diagnosed with an eating disorder?

No Yes, I have recovered Yes, I have not recovered

74. If so, what is/was your diagnosis?

- Anorexia nervosa
- Bulimia nervosa
- Eating Disorder Not Otherwise Specified (EDNOS)
- Binge eating disorder
- I don't know

75. If you have not been diagnosed with an eating disorder, do you feel you have had an eating disorder?

No Yes, in the past Yes, currently

76. If you have had an eating disorder, how old were you when your eating disorder began?

77. Are you currently in treatment for an eating disorder?

- No
- Yes, in outpatient care
- Yes, in a partial hospitalization program
- Yes, in a psychiatric residential facility
- Yes, in a medical hospital
- Other (Please Specify)

78. Have you been in treatment for an eating disorder in the past (check all that apply)?

- No
- Yes, in outpatient care
- Yes, in a partial hospitalization program
- Yes, in a psychiatric residential facility
- Yes, in a medical hospital
- Other (Please Specify)

79. If so, how old were you when you first got treatment for an eating disorder?

80. In the past 30 days were you medically hospitalized for an eating disorder?

Yes No

81. Have you ever been medically hospitalized for an eating disorder?
Yes No

82. If you have been medically hospitalized for an eating disorder, how many times were you hospitalized?

83. If you have been medically hospitalized, approximately how many total days have you spent in the hospital for an eating disorder?
Less than 30 days 30-60 days 60-120 days More than 120 days

84. Have you ever been diagnosed with low bone density, osteoporosis, or osteopenia?
Yes No

85. If you are a woman, have you ever missed menstrual periods due to having an eating disorder?
- No
- Yes, less than 3
- Yes, 3-6
- Yes, 6-12
- Yes, more than 12
- No, but I have been on the "pill"
- I have never had a period

86. If you are a woman, how many menstrual periods have you missed in the last year (other than for pregnancy)?
- None
- Less than 3
- 3-6
- 6-9
- 9-12
- None, but I have been on the "pill"
- I have never had a period

87. Have you ever been diagnosed with depression?
Yes No

88. Have you ever been diagnosed with an anxiety disorder?
Yes No

89. Have you ever been diagnosed with attention deficit hyperactivity disorder (ADHD)?
Yes No

90. Have you ever been diagnosed with any other psychiatric disorder (please specify)?

91. Have you ever been treated for any psychiatric disorder with medication?
No Yes, in the past Yes, currently

Disordered eating and other behaviors

92. Have you ever dieted to lose weight?
In the past Currently Never

93. How old were you when you first started dieting?

94. In the past 30 days did you count calories, fat grams or carbohydrate grams?
Yes No

95. Have you ever made yourself throw-up to control your weight?
Yes No

96. If so, how old were you when you first made yourself throw-up to control your weight?

97. During your life, for how many months have you made yourself throw-up?

- 0 months
- Less than 1 month
- 1-6 months
- 6-12 months
- 12-24 months
- More than 24 months

98. Have you ever binged (eaten what others would consider an unusually large amount of food for the circumstances, accompanied by a sense of having lost control over eating)?

Yes No

99. If so, how old were you when you first binged?

100. During your life, for how many months have you binged?

- 0 months
- Less than 1 month
- 1-6 months
- 6-12 months
- 12-24 months
- More than 24 months

101. Have you ever used laxatives to control your weight?

Yes No

102. If so, how old were you when you first used laxatives to control your weight?

103. During your life, for how many months have you used laxatives to control your weight?

- 0 months
- Less than 1 month
- 1-6 months
- 6-12 months
- 12-24 months
- More than 24 months

104. In the past 30 days did you use diet pills to control your weight?

Yes No

105. Have you EVER used diet pills to control your weight?

Yes No

106. If so, how old were you when you first used diet pills?

107. During your life, for how many months have you used diet pills?

- 0 months
- Less than 1 month
- 1-6 months
- 6-12 months
- 12-24 months
- More than 24 months

108. If you have used diet pills to control your weight, how often have you used diet pills?

- Less than 1 time/week
- 1 time/week
- 2-3 times/week

- 4-6 times/week
- 7 times/week or more

109. Have you ever exercised to control your weight?
In the past Currently Never

110. In the past 30 days did you harm yourself intentionally?
Yes No

111. Have you ever harmed yourself intentionally?
Yes No

112. If so, how have you harmed yourself?
- Cutting
- Burning
- Scratching
- Other/more than one (Please Specify)

Your activities

113. Are you currently in school?
Yes No

114. If you are in school, in the past 30 days, how many hours per week did you spend in school or on schoolwork?

115. Are you currently employed?
Yes No

116. If you are employed, in the past 30 days, how many hours per week did you spend working?

117. In the past 30 days how many hours per week did you spend hanging out with friends?

118. In the past 30 days how many hours per week did you spend in other extracurricular activities?

119. If you are in school, have you ever had to stop attending school because of an eating disorder?
Yes No

120. If you are working, have you ever had to stop working because of an eating disorder?
Yes No

121. In the past 30 days I spent ____ time on school/work due to having an eating disorder.
- A lot less time
- Some less time
- No change
- Some more time
- A lot more time

122. In the past 30 days I spent ____ time participating in extracurricular activities (sports, clubs, etc.) due to having an eating disorder.
- A lot less time
- Some less time
- No change
- Some more time
- A lot more time

123. In the past 30 days I spent ____ time with friends due to having an eating disorder.

- A lot less time
- Some less time
- No change
- Some more time
- A lot more time

124. In the past 30 days, if the time spent in any of the above activities has changed, how big of an impact did the change have on your life?

- No impact
- Small impact
- Medium impact
- Large impact
- Very large impact

Information about eating disorders

125. In the past 30 days where did you get information about eating disorders (check all that apply)?

- Parents
- Books
- Movies
- Friends
- Magazines
- Physicians
- Television
- School
- Newspaper
- Internet
- General health website
- Pro-recovery website
- Pro-ana website
- Pro-mia website
- Other (Please Specify) ____________

126. In the past 30 days where did you MOST OFTEN get information about eating disorders?

- Parents
- Books
- Movies
- Friends
- Magazines
- Physicians
- Television
- School
- Newspaper
- Internet
- General health website
- Pro-recovery website
- Pro-ana website
- Pro-mia website
- Other (Please Specify) ____________

Your internet usage

127. In the past 30 days about how much time, on average, did you spend on the internet every day?

Less than 1 hour 1-2 hours 2-4 hours More than 4 hours

Pro-Recovery websites

128. In the past 30 days, did you visit websites that promote recovery from eating disorders (pro-recovery

sites, for example www.somethingfishy.org)?

○ Yes ○ No

129. Have you ever visited pro-recovery sites?

○ Yes ○ No

If you have not visited these sites, skip to question 155.

130. If you have visited pro-recovery websites, which sites do you remember visiting?

131. How old were you when you first visited pro-recovery sites?

132. During your life, for how many months have you visited pro-recovery sites?

- ○ 0 months
- ○ Less than 1 month
- ○ 1-6 months
- ○ 6-12 months
- ○ 12-24 months
- ○ More than 24 months

133. How did you first learn about pro-recovery sites?

- ○ A friend
- ○ I read about them
- ○ I found them by chance
- ○ Weblog surfing (for example, Xanga, Livejournal)
- ○ Other (Please Specify)

134. In the past 30 days how often did you visit pro-recovery sites?

○ 1 time a month ○ 1 time a week ○ 1 time a day ○ more than 1 time a day

135. In the past 30 days how many hours did you spend on pro-recovery sites every week?

136. In the past 30 days from which locations did you access pro-recovery sites (check all that apply)?

- ☐ Home
- ☐ Parent's home
- ☐ Eating disorder treatment facility
- ☐ Work
- ☐ School
- ☐ Other (Please Specify)

137. In the past 30 days from which location did you MOST OFTEN access pro-recovery sites?

- ○ Home
- ○ Parent's home
- ○ Eating disorder treatment facility
- ○ Work
- ○ School
- ○ Other (Please Specify)

138. In the past 30 days for what reasons did you visit pro-recovery sites (check all that apply)?

- ☐ To maintain motivation for weight loss
- ☐ To learn weight loss tips
- ☐ To meet people
- ☐ To learn tips on how to hide your eating disorder
- ☐ Curiosity
- ☐ To find support

☐ Other (Please Specify) ______

139. In the past 30 days what was the MOST IMPORTANT reason you visited pro-recovery sites?

- ◯ To maintain motivation for weight loss
- ◯ To learn weight loss tips
- ◯ To meet people
- ◯ To learn tips on how to hide your eating disorder
- ◯ Curiosity
- ◯ To find support
- ◯ Other (Please Specify) ______

140. How motivational FOR RECOVERY are the photographs/artwork on pro-recovery sites?
◯ Extremely ◯ Very ◯ Somewhat ◯ A little ◯ Not at all

141. How motivational FOR RECOVERY are the forum postings/chat rooms on pro-recovery sites?
◯ Extremely ◯ Very ◯ Somewhat ◯ A little ◯ Not at all

142. How motivational FOR RECOVERY is the diet/nutrition/exercise information on pro-recovery sites?
◯ Extremely ◯ Very ◯ Somewhat ◯ A little ◯ Not at all

143. How motivational FOR RECOVERY are the diaries/journal entries/blogs on pro-recovery sites?
◯ Extremely ◯ Very ◯ Somewhat ◯ A little ◯ Not at all

144. In the past 30 days how supported did you feel by pro-recovery sites?
◯ Not at all ◯ A little bit ◯ Somewhat ◯ Very ◯ Extremely

145. Do you feel like you found a community of people like you on pro-recovery sites?
◯ Not at all ◯ A little bit ◯ Somewhat ◯ Very much ◯ Completely

146. In the past 30 days what did you do on pro-recovery sites (check all that apply)?

- ☐ Read posted information
- ☐ Visited a chat room
- ☐ Posted messages
- ☐ Learned about methods of weight loss or purging
- ☐ Learned about diet pills, laxatives, or weight loss supplements
- ☐ Read diaries/journal entries/blogs
- ☐ Learned about places to purchase diet pills, laxatives, or weight loss supplements
- ☐ Created my own diary/journal entry/blog

147. Have your eating habits changed as a result of visiting pro-recovery sites?
◯ Yes ◯ No

148. In the past 30 days did you use new diet pills, laxatives, or weight loss supplements as a result of visiting pro-recovery sites?
◯ Yes ◯ No

149. Have you EVER used new diet pills, laxatives, or weight loss supplements as a result of visiting pro-recovery sites?
◯ Yes ◯ No

150. In the past 30 days did you use new methods for weight loss or purging as a result of visiting pro-recovery sites?
◯ Yes ◯ No

151. Have you EVER used new methods of weight loss or purging as a result of visiting pro-recovery sites?
◯ Yes ◯ No

152. In the past 30 days did you harm yourself intentionally (e.g. cutting, scratching) as a result of visiting pro-recovery sites?
◯ Yes ◯ No

153. Have you EVER harmed yourself intentionally as a result of visiting pro-recovery sites?

Yes No

154. Do you have, or have you ever had your own pro-recovery site?
Yes No

Pro-Eating Disorder websites

155. In the past 30 days did you visit websites that promote eating disorders (pro-eating disorder, for example pro-ANA or pro-MIA sites)?
Yes No

156. Have you ever visited pro-eating disorder websites?
Yes No

If you have not visited these sites, skip to question 184.

157. If you have visited pro-eating disorder sites, which sites do you remember visiting?

158. How old were you when you first visited pro-eating disorder sites?

159. During your life, for how many months have you visited pro-eating disorder sites?
- 0 months
- Less than 1 month
- 1-6 months
- 6-12 months
- 12-24 months
- More than 24 months

160. How did you first learn about pro-eating disorder sites?
- A friend
- I read about them
- I found them by chance
- Weblog surfing (for example, Xanga, Livejournal)
- Other (Please Specify)

161. In the past 30 days about how often did you visit pro-eating disorder sites?
1 time a month 1 time a week 1 time a day More than 1 time a day

162. In the past 30 days how many hours did you spend on pro-eating disorder sites every week?

163. In the past 30 days from which locations did you access pro-eating disorder sites (mark all that apply)?
- Home
- Parent's home
- Eating disorder treatment facility
- Work
- School
- Other (Please Specify)

164. In the past 30 days from which location did you MOST OFTEN access pro-eating disorder sites?
- Home
- Parent's home
- Eating disorder treatment facility
- Work
- School
- Other (Please Specify)

165. In the past 30 days for what reasons did you visit pro-eating disorder sites (mark all that apply)?

- [ ] To maintain motivation for weight loss
- [ ] To learn weight loss tips
- [ ] To meet people
- [ ] To learn tips on how to hide your eating disorder
- [ ] Curiosity
- [ ] To find support
- [ ] Other (Please Specify) ________

166. In the past 30 days what was the MOST IMPORTANT reason you visited pro-eating disorder sites?

- ○ To maintain motivation for weight loss
- ○ To learn weight loss tips
- ○ To meet people
- ○ To learn tips on how to hide your eating disorder
- ○ Curiosity
- ○ To find support
- ○ Other (Please Specify) ________

167. How motivational FOR CONTINUING YOUR EATING DISORDER are the photographs/artwork on pro-eating disorder sites?

○ Extremely ○ Very ○ Somewhat ○ A little ○ Not at all

168. How motivational FOR CONTINUING YOUR EATING DISORDER are the forum postings/chat rooms on pro-eating disorder sites?

○ Extremely ○ Very ○ Somewhat ○ A little ○ Not at all

169. How motivational FOR CONTINUING YOUR EATING DISORDER is the diet/nutrition/exercise information on pro-eating disorder sites?

○ Extremely ○ Very ○ Somewhat ○ A little ○ Not at all

170. How motivational FOR CONTINUING YOUR EATING DISORDER are the diaries/journal entries/blogs on pro-eating disorder sites?

○ Extremely ○ Very ○ Somewhat ○ A little ○ Not at all

171. In the past 30 days how supported did you feel by pro-eating disorder sites?

○ Not at all ○ A little bit ○ Somewhat ○ Very ○ Extremely

172. Do you feel like you found a community of people like you on pro-eating disorder sites?

○ Not at all ○ A little bit ○ Somewhat ○ Very much ○ Completely

173. In the past 30 days what did you do on pro-eating disorder sites (mark all that apply)?

- [ ] Read posted information
- [ ] Visited a chat room
- [ ] Posted messages
- [ ] Learned about methods of weight loss or purging
- [ ] Learned about diet pills, laxatives, or weight loss supplements
- [ ] Read diaries/journal entries/blogs
- [ ] Learned about places to purchase diet pills, laxatives, or weight loss supplements
- [ ] Participated in point counting challenges
- [ ] Created my own diary/journal entry/blog

174. Have your eating habits changed as a result of visiting pro-eating disorder sites?

○ Yes ○ No

175. In the past 30 days did you use new diet pills, laxatives or weight loss supplements as a result of visiting pro-eating disorder sites?

○ Yes ○ No

176. Have you EVER used new diet pills, laxatives or weight loss supplements as a result of visiting pro-eating disorder sites?

○ Yes ○ No

177. In the past 30 days did you use new methods of weight loss or purging as a result of visiting pro-eating disorder sites?
○ Yes ○ No

178. Have you EVER used new methods for weight loss or purging as a result of visiting pro-eating disorder sites?
○ Yes ○ No

179. In the past 30 days did you harm yourself intentionally (e.g. cutting, scratching) as a result of visiting pro-eating disorder sites?
○ Yes ○ No

180. Have you EVER harmed yourself intentionally as a result of visiting pro-eating disorder sites?
○ Yes ○ No

181. Do you have, or have you ever had your own pro-ANA or pro-MIA site?
○ Yes ○ No

182. Do you support the pro-eating disorder (pro-ana, pro-mia, etc.) movement?
○ Not at all ○ A little bit ○ Somewhat ○ Very much ○ Completely

183. Do you have a pro-ana red bracelet?
○ Yes ○ No

Self Injury websites

184. In the past 30 days did you visit self-injury websites?
○ Yes ○ No

185. Have you EVER visited self-injury sites?
○ Yes ○ No

186. If you have visited self-injury sites, in the past 30 days did you harm yourself intentionally (e.g. cutting, scratching) as a result of visiting these sites?
○ Yes ○ No

187. If you have visited self-injury sites, have you EVER harmed yourself intentionally as a result of visiting these sites?
○ Yes ○ No

Self-View

188. In the past 30 days how did you view yourself with regard to your eating?
- ○ Healthy
- ○ Sick
- ○ Recovering
- ○ Trying to recover
- ○ Eating disordered by choice
- ○ Other/more than one (Please Specify) ________

189. Do you see eating disorders as a lifestyle choice?
○ Yes ○ No

190. What role do you think pro-recovery and pro-ANA/pro-MIA websites played in the course of your eating disorder or in your life?

191. Did you lie on any of your responses in this survey?
◯ Yes ◯ No

192. If so, what did you lie about?

☐ My age

☐ My current weight

☐ Other (Please Specify)

193. Use this space to clarify any of your answers, let us know what you thought about the survey, or to provide us with any other information.

This Survey is NOT yet functional

© 2006 Stanford University School of Medicine Departments Directions & Maps Contacts Site by IRT Terms of Use
